# Cost-sharing strategies combining targeted public subsidies with private-sector delivery achieve high bednet coverage and reduced malaria transmission in Kilombero Valley, southern Tanzania

**DOI:** 10.1186/1471-2334-7-121

**Published:** 2007-10-25

**Authors:** GF Killeen, A Tami, J Kihonda, FO Okumu, ME Kotas, H Grundmann, N Kasigudi, H Ngonyani, V Mayagaya, R Nathan, S Abdulla, JD Charlwood, TA Smith, C Lengeler

**Affiliations:** 1 Ifakara Health Research and Development Centre, Box 53, Ifakara, Morogoro, United Republic of Tanzania; 2 Department of Public Health and Epidemiolology, Swiss Tropical Institute, Socinstrasse 57, Basel, CH 4002, Switzerland; 3 School of Biological and Biomedical Sciences, Durham University, South Road, Durham, DH1 3LE, UK; 4 KIT (Royal Tropical Institute), Biomedical Research, Meibergdreef 39, 1105 AZ Amsterdam, The Netherlands; 5 Faculty of Health Sciences, Moi University, P.O Box 4606, Eldoret, Kenya; 6 Department of Biomedical Engineering, Yale University, P.O. Box 208284; New Haven, CT 06520-8284, USA; 7 Centre for Infectious Diseases Epidemiology, National Institute for Public Health and the Environment (RIVM), PO Box 1, 3720 BA Bilthoven, The Netherlands; 8 Danish Bilharziasis Laboratories, Jaegersborg Allé 1-D, Charlottenlund, DK 2920, Denmark

## Abstract

**Background:**

Cost-sharing schemes incorporating modest targeted subsidies have promoted insecticide-treated nets (ITNs) for malaria prevention in the Kilombero Valley, southern Tanzania, since 1996. Here we evaluate resulting changes in bednet coverage and malaria transmission.

**Methods:**

Bednets were sold through local agents at fixed prices representing a 34% subsidy relative to full delivery cost. A further targeted subsidy of 15% was provided to vulnerable groups through discount vouchers delivered through antenatal clinics and regular immunizations. Continuous entomological surveys (2,376 trap nights) were conducted from October 2001 to September 2003 in 25 randomly-selected population clusters of a demographic surveillance system which monitored net coverage.

**Results:**

Mean net usage of 75% (11,982/16,086) across all age groups was achieved but now-obsolete technologies available at the time resulted in low insecticide treatment rates. Malaria transmission remained intense but was substantially reduced: Compared with an exceptionally high historical mean EIR of 1481, even non-users of nets were protected (EIR [fold reduction] = 349 infectious bites per person per year [×4]), while the average resident (244 [×6]), users of typical nets (210 [×7]) and users of insecticidal nets (105 [×14]) enjoyed increasing benefits.

**Conclusion:**

Despite low net treatment levels, community-level protection was equivalent to the personal protection of an ITN. Greater gains for net users and non-users are predicted if more expensive long-lasting ITN technologies can be similarly promoted with correspondingly augmented subsidies. Cost sharing strategies represent an important option for national programmes lacking adequate financing to fully subsidize comprehensive ITN coverage.

## Background

The efficacy of insecticide-treated nets (ITNs) for preventing malaria is well established [1,2] and they are considered to be one of the most promising interventions for large-scale implementation in Africa [3-5]. While the merits of various distribution systems have proven contentious in recent years [6,7], a variety of market-based, public-sector and hybrid distribution systems for ITNs [8-12] have emerged which merit investigation, development and comparative evaluation on scales for which no precedent yet exists [3]. Even the most recent review [13] highlights that the existing evidence base is not sufficient to enable rational choice of specific strategies for subsidization and delivery. Nevertheless, consensus is emerging that coverage targets for ITNs should be revised to maximize public health impact by reducing malaria transmission in entire populations rather than merely providing personal protection to those most at risk [5,14,15].

A major challenge to National Malaria Control Programmes (NMCPs) across Africa is to achieve and measure community-level or mass effects of nets in addition to the individual protection offered to those actually sleeping under one [5,14,16]. ITNs protect not only the individuals and households that use them, but also members of the surrounding community [16-23]. This is because they kill adult mosquitoes directly or force them to undertake longer, more hazardous foraging expeditions in search of vertebrate blood and aquatic habitats [24-27]. While this mass effect has been demonstrated repeatedly by efficacy trials with high coverages of nets [16,17,21,28], they have also been demonstrated under effective programmatic conditions [20,23]. Theoretical [14] and experimental studies [16,23] have suggested that communal protection resulting from moderate coverage levels in entire populations may be at least as important as the personal protection achieved through targeted delivery to vulnerable groups such as pregnant women and young children [29,30]. This has substantial equity implications since the mass effect may protect entire communities, including the most vulnerable who cannot access or use an ITN. Achieving and measuring these community-level effects and their dependence on ITN coverage on scales large-enough to be representative remains notoriously difficult [31] but is nevertheless essential for planning national control programmes [14]. There is therefore an urgent need to evaluate the effectiveness of ITN's in a large-area trials under realistic programmatic conditions [32] where the distribution of nets is heterogeneous but not experimentally controlled and both treated and untreated nets co-exist under representative conditions of availability, use and maintenance. As whole-population coverage with nets and community-level suppression of transmission are now increasingly prioritized [5,14,15], the most important remaining question is how these goals can be attained and sustained with the growing but finite financial resources available to NMCPs in Africa [14,15,33].

Given such challenging and comprehensive coverage targets for a commodity worth several days income to an impoverished rural African family, it is hardly surprizing that free or highly subsidized provision of ITNs is the preferred option of NMCPs and international agencies alike [5,15]. Comprehensive subsidization up to the level of provision at no cost to the user may be particularly useful for "catching up" to defined coverage targets which may then be sustained with more modest subsidies [8,12,34]. While the global economy can certainly afford such investment in the health of its poorest citizens, such comprehensive international commitment to financing ITNs has yet to be realized [15,33]. Major investments by the Global Fund to Fight Aids, Tuberculosis and Malaria and the United States President's Malaria Initiative have now made substantial financing available to National Malaria Control Progammes (NMCPs) across Africa. Sadly, even these donations are inadequate and currently support only a fraction of the full cost of providing ITNs to at-risk populations in Africa [15,33]. Unless African NMCPs can secure the $1.7–2.2 billion they need to control malaria each year [33], cost-sharing schemes for ITN distribution will remain an essential strategic option [3,14].

**Figure 1:**
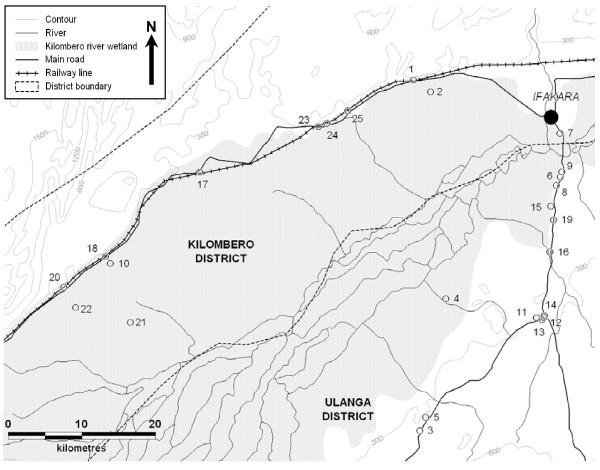
Map of the Kilombero Valley and the 25 sampling clusters described in table 1.

Although cost-sharing approaches to ITN distribution face substantial challenges [11,35-37], notable success in terms of coverage and impact have been reported in a variety of settings [9,12,34,38,39] including the Kilombero Valley in southern Tanzania (Figure 1) where ITNs have been promoted and subsidized since 1996 [11,23,35,40-49]. Much of the essential experience generated by KINET, using discount vouchers to target limited subsidies at vulnerable groups, was later integrated into the ITN promotion strategy of the National Malaria Control Programme of the United Republic of Tanzania [35,50,51].

Here we present a detailed entomological evaluation of a large-scale study of the well-established social marketing programmes for bednets in the Kilombero Valley, southern Tanzania. Specifically, this study was implemented to identify key determinants of human exposure, to evaluate the level of coverage achieved, and to measure the overall impact on mosquito populations and malaria transmission intensity.

## Methods

### Study area

The epidemiology of malaria in the Kilombero Valley has been well described and a number of malaria control interventions have been evaluated in this setting, notably the KINET social marketing program for subsidizing and promoting bednets use [11,23,35,40-49]. The malaria transmission systems of this valley, and the village of Namwawala in particular, have been well characterized, [52-69]. This low-lying, flooding river valley has historically experienced very high transmission intensities, including the highest reported EIR we are aware of, with the occupants of one house experiencing an estimated 2,979 infectious bites per year in the early 1990s [54].

### Bednet promotion and subsidization

The bednet promotion strategy implemented from 1996 onwards has been described [40] and evaluated [11,23,35,41-49] in considerable detail elsewhere but is outlined briefly as follows. Following careful sensitization and market research within the Kilombero Valley, a generic branding and price-fixing system was developed for marketing a limited number of recommended insecticide and net products. These endorsed products were commonly branded under the name *Zuia Mbu*, literally meaning "Prevent mosquitoes" in Kiswahili, and distributed through a range of contracted private and public sector agents chosen by the communities themselves. The retail price of nets was fixed at Tsh 3000 or approximately US$5 at the time, corresponding to a cost recovery on total distribution costs of 66% with the balance reflecting a 34% public subsidy on a typical net. Further subsidy was provided to vulnerable pregnant women and infants by providing discount vouchers for each qualifying individual at antenatal clinics and routine immunizations, respectively. The voucher entitled the recipient to a discount of Tsh 500 (approximately US$0.84 at the time) on *Zuia Mbu *nets purchased through the contracted agents described above. This represents an additional 15% subsidy targeted specifically at vulnerable population groups amongst whom the benefits of personal protection are most important.

### Sampling frame for entomological collection to estimate of malaria transmission intensity

An important aspect of the study was that the primary sampling units were not areas but individuals and their households. These were selected randomly from the database of the Demographic Surveillance System (DSS) of the Ifakara Health Research and Development Centre [41], which at the time included approximately 65,000 individuals in circa 16,000 households, distributed into 25 villages (*Vijiji*) and 105 subvillages (*Vitongoji*) in the two districts of Kilombero and Ulanga [41]. Subvillages were stratified by district (Kilombero or Ulanga) and into five strata of mosquito net coverage per household, as determined from the 2000 Social and Economic Survey (SES). Subvillages were sampled within these strata, with sampling probabilities proportionate to the number of households in the SES, resulting in 14 of the sampled 25 subvillages being in Ulanga district because there were no villages in Kilombero in the two highest categories of mosquito net coverage.

The sampling strategy aimed at defining small clusters of 12 houses around (and including) an 'index' house determined by the individual designated as household head. The 25 selected subvillages were assigned at random to weeks and were visited on a 25-week cycle at 6 month intervals over a two year period (October 2001–September 2003). Within each subvillage, 10 selected households were listed in random order and CDC light traps were assigned to a specific index individual within the first consenting household that could be recruited in their order in these lists. Where the identified person was sleeping in a farm (*shamba*) house or shelter, this is where mosquito sampling occurred. The houses sampled during each 2 day period comprised this index person house and its immediate neighbours. The nearest house to the index house was used for bed net collections, the next nearest house was assigned the second light trap and so on until six bednet and six light trap collections were assigned. These houses and nets were sampled for two consecutive days. In the last two days of the weekly routine, a new index person was recruited within the same subvillage by selecting from the same list in order of appearance. Replication every six months used the same lists of index persons and population sampling clusters. The location of these sampling clusters, sorted by village and subvillage are listed in Table 1 and illustrated in Figure 1. In each sampled house, on each occasion, a questionnaire was administered to assess characteristics of house construction, the number of people sleeping in the house with and without nets, the quantity and quality of nets in the house and the times that residents awoke in the morning and went to bed in the evening [69].

**Table 1:** Median^a ^locations of population clusters sampled for mosquitoes within the Ifakara Health Research and Development Centre's demographic surveillance system (Figure 1).

Cluster	Village (*Kijiji*)	Subvillage (*Kitongoji*)	Coordinates (degrees)	
			
			Latitude (S)	Longitude (E)
				
1	Idete	Godawn	36.510350	8.098190
2		Msumbiji	36.531390	8.112070
3	Iragua	Igunda	36.517925	8.525285
4	Kichangani	Mahongole	36.550050	8.259690
5	Kidugalo	Kidugalo	36.525490	8.509210
6	Kivukoni	Butiama	36.691260	8.216390
7		Chikago	36.690630	8.162715
8		Gezaulole	36.686410	8.226495
9		Ramba	36.693365	8.209740
10	Lukolongo	Lukolongo	36.136540	8.321935
11	Lupiro	Libaratula	36.661665	8.387570
12		Lupiro Kati	36.671240	8.387535
13		Madibila	36.668540	8.390000
14		Ndoro	36.671600	8.385095
15	Mavimba	Manjole	36.679735	8.252015
16		Manjole	36.678285	8.307580
17	Mbingu	Mbingu	36.246770	8.210105
18	Mchombe	Mchombe	36.130575	8.313275
19	Minepa	Kisakimbali	36.682795	8.268375
20	Mkangawalo	Itongoa A	36.079015	8.350290
21		Mgudeni	36.161390	8.393270
22		Mkangawalo	36.093330	8.375495
23	Namwawala	Namwawala A	36.393005	8.154425
24		Namwawala B	36.403520	8.150930
25		Videnge	36.428950	8.135285

^a^Note that because several houses were surveyed and several index individuals moved several times during the study, some locations appear inconsistent with their allocation to s specific village.

### Mosquito collection and processing

In addition to the those in the six houses selected for light trap sampling on each night, all nets in the houses of an additional six individuals were searched for mosquitoes each morning using standard aspirators to collect them [70]. Centers for Disease Control (CDC) light traps were placed as close as possible to occupied nets at a height of approximately 50 cm, as previously described [71,72] except that an enlarged catch net was used in which water was provided to mosquitoes so as to minimize mortality during collection. We made no attempt to differentiate between treated and untreated nets in the field as this is impractical during routine field surveys and insecticide treatment has only a minor effect on sampling efficiency [73]. On occasions when the selected individual for light trap sampling lacked a net, he or she was provided with an untreated net for the nights during which they participated.

All mosquitoes were first identified to sex and species based on morphological criteria and then classified visually as being unfed, partially fed, fed or gravid [74,75]. Sporozoite infection prevalence was determined by circumsporozoite protein ELISA [76] using pools of 10 or fewer mosquitoes, from which each positive reaction was assumed to represent only one infected mosquito. For each sampling cluster from which sufficient numbers of *An. gambiae sensu lato *were obtained, the sibling species identity of 50 individual mosquitoes were determined by polymerase chain reaction [77].

### Calibration of CDC light traps to estimate exposure of humans to mosquito bites

The sampling efficiency of the CDC light trap was estimated using a 3 × 3 Latin square design to compare the CDC light trap with a human landing catch gold standard and an Mbita bednet as an alternative, as previously described [78,79], respectively. Over the course of 23 nights (8 rotations of 3 nights in 3 randomly selected houses in cluster 11, minus one night during which work was cancelled for logistical reasons) of indoor sampling, the human landling catch, CDC light trap and Mbita bednet trap caught a total of 2477, 1005 and 37 *Anopheles gambiae sensu lato*, 45, 41 and 3 *An. funestus *and 172, 136 and 8 *Culex *species per night. Given that 90% of transmission in this setting was observed to occur indoors during parallel studies in the same location at the same time [69] and CDC light trap catches are typically directly proportional to human landing catches [78-82], we consider indoor sampling with CDC light traps to be approximately representative of true adult human exposure, with sampling efficiencies for each species equivalent to the quotient of its mean catch respective to that of the human landing catch, adjusted for the fact that human landing catch was conducted for only 45 minutes (75%) of each hour.

The level of personal protection afforded by ITNs against *An. gambiae s.l*. was estimated based on all-night indoor and outdoor human landing catches in cluster 11, combined with estimates of personal protection against indoor exposure determined from experimental hut trials [69]. While insufficient data was available for any other mosquito species or genus, only 10% of exposure was estimated to occur outdoors in this setting and a reasonably well maintained ITN is estimated to protect against 70% of infectious bites from this vector [69].

### Household and individual determinants of mosquito density and light trap sensitivity

In order to identify household risk factors for exposure to transmission, and to confirm that CDC-light traps are indeed a reliable sampling tool regardless of the insecticidal properties of nets, we evaluated the effects of such determinants upon the numbers of mosquitoes caught in these traps. The analysis of factors associated with houses upon mosquito density was complicated by the repeated sampling of the same houses on consecutive days within one round or on separate rounds. The influence of each factor on mosquito density (B) was therefore determined by fitting a mixed model to the values of log (B+1) with first order autoregressive covariance in the community level variance associated with sampling any given cluster during a given round, while repeated sampling of individual house structures was treated as a random factor. All other factors and covariates were treated as fixed factors. Initially all factors associated with the house in which the sample was obtained were included in the model and the model was refined by backward elimination of variables until only significant (P ≤ 0.05) ones remained.

### Spatial and temporal heterogeneity of transmission dynamics

Malaria transmission in the valley was observed to be highly seasonal and each cluster could only be sampled twice a year so frequent longitudinal samples were not obtained for each cluster. We therefore estimated cluster-specific estimates of biting rate and sporozoite rate by comparing direct estimates for each cluster with an expected valley-wide mean for that point in time, obtained by temporal smoothing with centred moving averages of estimates from all the clusters. Consistent differences between direct and smoothed estimates for each cluster were estimated with mixed models and used to estimate cluster-specific mean biting and sporozoite rates so that local annual EIR could be calculated.

First the crude EIR estimate for the two major species were refined by multiplying the smoothed biting rate (B) estimates by concurrent smoothed sporozoite rate estimates. The relative population densities of mosquitoes in the 25 sampling clusters were then estimated by fitting mixed models of difference between the log (B+1) transformed crude biting rate estimates and corresponding smoothed estimates. Cluster was treated as a fixed factor while round was treated as a repeated measure in a first order autoregressive model. The resulting odds ratios were used to adjust the overall valley wide EIR estimate for each species in proportion to the estimated relative biting density for that cluster. We also attempted to estimate cluster-specific heterogeneities of sporozoite rates (S) using the same approach but transforming this binary outcome to convert it into the approximately normally-distributed dependent function *arcsine*(S^0.5^).

### Comparison of recent EIR measurements with historical precedents and expected values

All available literature describing malaria transmission in the Kilombero was reviewed and estimates of human biting rates, sporozoite prevalences and EIR were tabulated for comparison with the recently-measured values reported here. As previous records had been recorded and analyzed by village, the recent data was also aggregated to village level to allow direct comparison where possible. All biting rates were recalculated using sampling efficiency estimates obtained as described above (see results), rather than the figure of 0.66 [72] as previously described [54,55]. Furthermore, biting rates were re-calculated as absolute annual means, rather than William's means as had has previously been reported[65]. This approach is consistent with that used to generate the more recent estimates presented above, the most recent commonly agreed definitions [83,84], and the true total exposure of humans which includes the higher values in over-dispersed data accounting for the bulk of transmission [84,85]. Note that this approach is nevertheless consistent with the log transformations used in earlier sections because these represent logarithms of mean biting densities rather than means of the logarithms of individual measurements. Note that all previous reports from Kilombero and Ulanga districts were included in this comparison except for the two villages of Michenga [86] and Kibaoni [53] which were not included in this study, as well as two reports from Ifakara town [52,87] which are considered urban or peri-urban and therefore cannot be rationally compared with any of the other sites surveyed [88-90].

In order to compare our observations with reasonable expectations, the impact of increasing coverage of bednets on malaria transmission was simulated assuming a plausible range of personal protection properties for bednets now and in the future. The effect of bednets upon human biting rate, sporozoite prevalence and entomological inoculation rate of *An. gambiae *was modelled as previously described [91] but with the following adaptations to this particular application. The study area is dominated by a mixture of zoophagic *An. arabiensis *and anthropophagic *An. gambiae *and diversion to alternative hosts can greatly influence the impacts of bednets [14,91] so we set the availability of individual cattle to a value of 0.8 × 10^-3 ^successful feeds per day per host-seeking mosquito per cow, representing an approximate mean of the values for these two species weighted according to their relative abundance as determined in these surveys. The influence of such alternative hosts was considered by simulating a village population of 1000 humans and 100 cattle, approximately consistent with demographic and agricultural trends in the study area. The biodemographic properties of the vector and sporogonic-stage parasite populations were modelled over coverage levels varying from 0 to 95% usage, corresponding to reported historical norms and an ideal future scenario, respectively. Similarly, the protective insecticidal (μ_p_) and diversionary properties (Δ_p_) of bednets were varied from 0.1 to 0.8, reflecting the most pessimistic estimates of mean condition and treatment level [92] through to the ideal properties of the most recently developed and evaluated long-lasting technologies [93-95]. At the four levels of protection considered (μ_p _= Δ_p _= 0.1, 0.2, 0.4 and 0.8), such bednets are expected to protect against 19, 36, 64 and 96% of indoor exposure, as estimated in the experimental hut trials typically used to evaluate such technologies [93-95]. To enable comparison with historical and recent reports from the Kilombero valley, our existing transmission models, largely parameterized in the village of Namwawala, were used to calculate relative changes in human biting rate, sporozoite rate and EIR as bednet coverage increases and scaled to their mean historical values in the study area. Note that a full set of suitable parameter estimates for *An. funestus *are not available so malaria transmission by this species was not simulated.

## Results

### Valley wide transmission intensity

**Figure 2:**
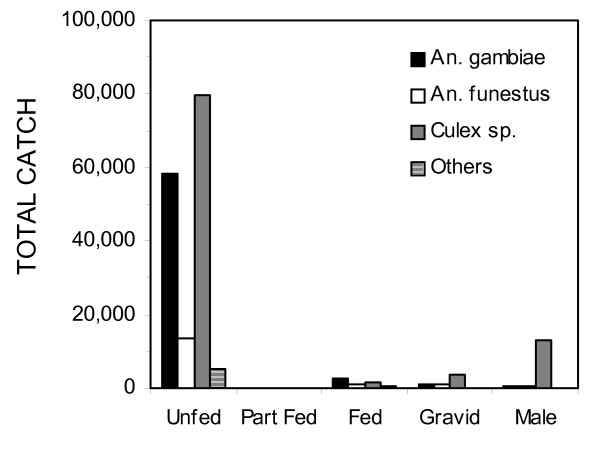
The distribution of sex and physiological status of mosquitoes caught in CDC light traps during the course of the study.

**Figure 3:**
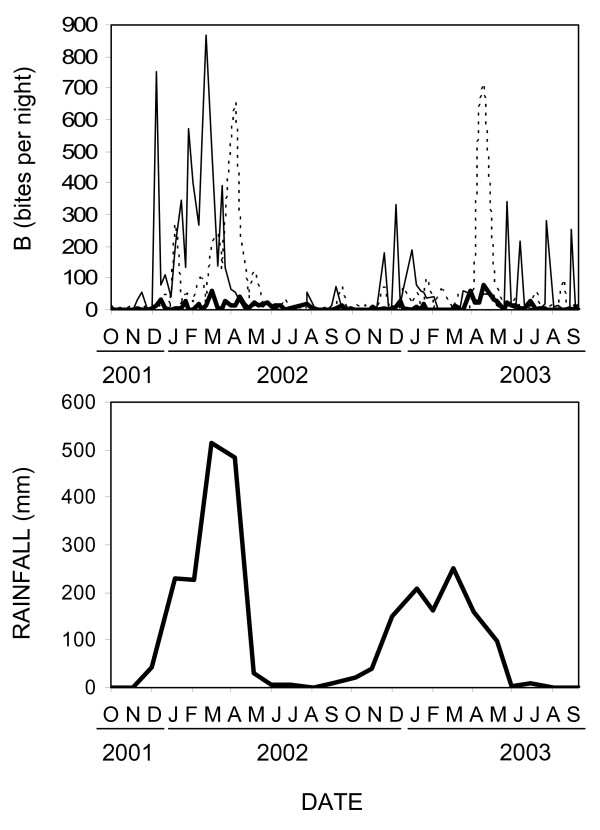
Crude biting rate estimates for mosquitoes (B) and monthly rainfall measurements in the Kilombero Valley during the study period. Thin solid line: *An. gambiae*; thick solid line: *An. funestus*; thin dotted line: *Culex *species.

Overall, 2,376 successful CDC light trap nights of sampling were conducted over the two year period of the study. Over fourteen thousand male mosquitoes, the vast majority of which were culicines, were trapped and discarded. The remaining bulk of the catch were female mosquitoes, most of which were unfed and therefore appear to have been host-seeking (Figure 2). Of these 62404 were *An. gambiae sensu lato*, 15840 were *An. funestus*, 85157 were *Culex *sp. and 5889 consisted of various other mosquito species, including anophelines thought to play little or no role in malaria transmission within the Kilombero Valley [66]. Calibration of the light trap method estimated sampling efficiencies, relative to human landing catch, of 0.30, 0.68 and 0.59 for *An. gambiae s.l*., *An. funestus *and *Culex *species, respectively. Using the same analytical methodology as previously applied to light trap catch data from this valley [54,55] and these estimated sampling efficiencies, we estimate crude mean biting rates for the entire study area over the two year study period of 62.5, 7.0 and 43.2 bites per person per night for *An. gambiae*, *An. funestus *and *Culex *sp., respectively. The overall sporozoite prevalence rates in the valley were estimated to be 0.98% (165/16910) for *An. gambiae *and 1.67% (122/7333) for *An. funestus*, with the two species differing significantly (P = 5.4 × 10^-6 ^by Χ^2 ^test). Thus the crude estimates of mean transmission intensity for the valley are therefore 307, 59 and 366 infectious bites per person per year for *An. gambiae*, *An. funestus *and the two species combined, respectively. The overall approximate dependence of mosquito population dynamics on rainfall can be seen in Figure 3. Most of the *An. gambiae *caught were caught in the main wet season of early 2002. *An. funestus *populations were generally much lower with similar seasonal peaks while *Culex *species peaked two months after *An. gambiae *in early 2002.

### Seasonality and spatial heterogeneity of malaria transmission

**Figure 4:**
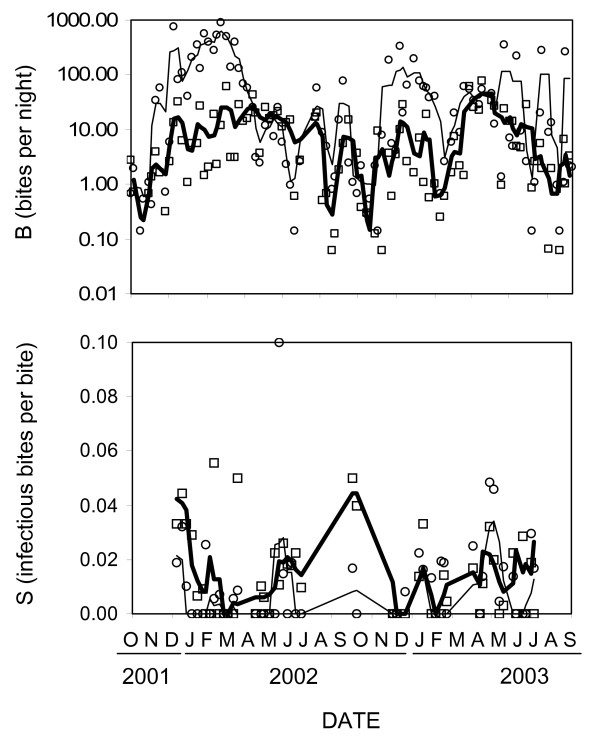
Smoothed mean biting (B) and sporozoite prevalence (S) of malaria vectors and the corresponding crude measurements in each cluster sample throughout the study. Thin solid line and circles: *An. gambiae*; thick solid line and squares: *An. funestus*

Figure 4 illustrates seasonal fluctuations in biting and sporozoite rates. Estimating EIR based on the smoothed, rather than crude, estimates of biting rate and sporozoite rate results in essentially identical EIR estimates of 310, 60 and 370 for *An. gambiae s.l*., *An funestus *and the two combined, respectively. The 25 sampling clusters showed considerable consistent heterogeneities of biting rate, with the effect of cluster approaching significance for *An. gambiae *and being highly significant for *An. funestus *(numerator d.f. = 25, denominator d.f. = 5.84, F = 3.25, P = 0.076 for *An. gambiae *and numerator d.f. = 25, denominator d.f. = 17.46, F = 5.71, P < 0.001 for *An. funestus *by mixed linear modelling as described in methods). In contrast, no significant heterogeneity of sporozoite rate could be detected between the sampling clusters (numerator d.f. = 21, denominator d.f. = 6.17, F = 0.50, P = 0.892 and numerator d.f. = 25, denominator d.f. = 0, F = 0.001, P not estimable, for *An gambiae *and *An. funestus*, respectively). We therefore estimated cluster-specific EIR values based on the valley-wide EIR for each vector and the relative biting rate of that cluster for that species (Table 2). Although locally adjusted EIR values vary widely, transmission intensity was ubiquitously high. The lowest and highest estimated EIRs for non-users of ITN were 75 and 1127 infectious bites per year, respectively, with the average across the clusters being 352 infectious bites per year. No significant difference was observed between the sporozoite prevalence in different villages (numerator d.f. = 21, denominator d.f. = 6.17, F = 0.498, P = 0.892 for *An. gambiae *and numerator d.f. = 25, denominator d.f. = 0, F = 0.001, P inestimable for *An. funestus*). Cluster-adjusted EIR for *An. gambiae *was not correlated to the proportion of *An. gambiae s.s*. comprising the *An. gambiae s.l*. population in each sampling cluster (CC = 0.220, 0.336 and CC = -0.155, P = 0.527, respectively by Pearson's correlation and Spearman's rank correlation).

**Table 2:** Net Coverage and locally adjusted estimates of entomological inoculation rates for each sampling cluster.

Cluster	Net coverage				Proportion *An. gambiae sensu stricto*.^c^	Entomological Inoculation Rate (Infectious bites per person per year)			
			
	Ownership^a^		Usage^b^			ITN non-users^d^			ITN Users^e^
					
	(%)	(n)	(%)	(n)		*An. gambiae*	*An. funestus*	Total	Total
									
1	44.0	505	82.8	505	ND	103	83	186	56
2	41.5	514	90.3	444	ND	466	49	515	154
3	36.8	1266	58.3	1249	0.71	1059	67	1127	338
4	26.9	766	48.2	711	0.66	206	40	245	74
5	46.5	1460	53.9	1664	ND	69	93	162	49
6	48.8	790	81.0	797	0.92	115	59	174	52
7	48.2	1008	78.0	1021	0.96	146	16	163	49
8	39.2	409	82.1	409	0.92	191	23	214	64
9	43.1	657	83.5	669	0.69	215	33	248	75
10	38.4	654	86.5	654	ND	358	56	414	124
11	48.7	739	75.9	739	0.37	372	102	474	142
12	52.5	610	85.7	618	0.04	624	85	709	213
13	43.5	170	86.5	177	0.47	568	68	636	191
14	53.9	1283	86.6	1283	0.31	797	54	851	255
15	20.8	454	85.5	469	0.66	383	60	444	133
16	41.8	539	78.3	540	0.85	96	51	147	44
17	44.4	563	73.3	677	0.28	192	20	212	64
18	37.2	332	82.2	332	ND	247	45	291	87
19	41.3	24	77.5	282	0.70	55	20	75	22
20	37.6	477	83.5	478	ND	26	54	80	24
21	12.1	685	30.2	701	0.65	420	29	449	135
22	28.4	263	70.0	263	ND	45	40	85	25
23	42.4	527	70.6	576	0.64	48	52	100	30
24	32.7	1242	72.1	849	ND	404	52	456	137
25	27.1	149	59.9	149	0.53	282	51	333	100
									
Mean	39.1	643	74.5	650	0.61	299	52	352	105

^a ^Expressed as the proportion of people sleeping under nets and derived from socioeconomic surveys which included number of members and nets in each household.

^b ^Expressed as the number of nets per person and derived from routine demographic surveillance system questionnaires which included the numbers of people who slept and slept under a net in each household.

^c ^Estimated by PCR analysis of *An. gambiae sensu lato *[77] for all clusters from which 50 samples could be successfully amplified. All samples which were not determined to be *An. gambiae sensu stricto *were identified as *An. arabiensis*. ND; Not determined because insufficient numbers of samples (n = 50 in all clusters for which an estimate is available) could be were obtained and successfully amplified.

^d ^Estimated by fitting mixed linear models of the difference between the log (x+1) of immediate and centres moving averages of biting density (See figure 4), treating cluster as a fixed effect and sample round as a repeated effect (see methods).

^e ^Calculated as the total EIR experienced by non-users in each cluster adjusted for the estimated 70% personal protection ITNs provide against exposure in Kilombero [69].

### Household and individual determinants of mosquito density and light trap sensitivity

The characteristics of the sampled houses and their influence on mosquito density are described in Table 3. Curiously, the 14 farm or *shamba *houses sampled with light traps appear to have considerably lower densities of *An. gambiae s.l*. than main residences in the same *kitongoji*. However, given that 13 of these houses or 58 of the 59 observations occurred in one cluster (number 4), this is likely to be a chance feature of local ecology, such as proximity to larval habitats, and probably not generalizable to the valley as a whole. Consistent with previous reports [55], the presence of neither unprotected nor screened windows had any influence on the indoor biting density of *An. gambiae s.l*. but houses with open eaves had considerably higher densities of *An. gambiae s.l*., consistent with the eaves being the primary point of house-entry by this species [74,96]. No structural feature other than wall construction influenced the measured density of *An. funestus *and the density of *Culex *sp. appeared to be largely independent of house structure. The reason for higher densities of *An. funestus *in houses with brick walls is difficult to interpret but suggests increased vulnerability or attractiveness of such houses to this species because of house entry or indoor resting preferences.

**Table 3:** Characteristics of the houses sampled for mosquitoes and their influence on light trap catches of mosquitoes.

Characteristic	Crude Frequency^a^		Influence on light traps catches^b^					
		
			*An. gambiae*		*An. funestus*		*Culex*	
					
	N	%	Odds ratio [95% CI]	P	Odds ratio [95% CI]	P	Odds ratio [95% CI]	P
								
*Function*				***0.002***	*NS*	*0.350*	*NS*	*0.105*
Main residence	2450	97.6	**1.00**	**NA**				
Farm (*shamba*) house	59	2.4	**0.30 [0.15–0.61]**	**0.001**				
								
*Walls*			*NS*	*0.318*		***0.057***	*NS*	*0.113*
Mud	1761	70.2			**1.000**	**NA**		
Bricks	706	28.1			**1.259 [1.101–1.428]**	**0.001**		
Other	42	1.7			**ND**	**ND**		
								
*Roof*			*NS*	*0.207*	*NS*	*0.701*	*NS*	*0.582*
Thatch	1920	76.5						
Corrugates iron	589	23.5						
								
*Eaves*				***0.002***	*NS*	*0.134*	*NS*	*0.214*
Closed	256	10.2	**1.00**	**NA**				
Open	2253	89.8	**1.54 [1.23–1.93]**	**<0.001**				
								
*Windows*			*NS*	*0.763*	*NS*	*0.692*	*NS*	*0.422*
None	402	16.0						
Open	2001	79.8						
Closed	106	4.2						
								
*Foundation*			*NS*	*0.815*	*NS*	*0.778*	*NS*	*0.112*
Raised on poles	22	0.9						
Built on ground	2487	99.1						
								
*Kitchen*			*NS*	*0.060*	*NS*	*0.205*	*NS*	*0.088*
Food cooked indoors	1016	40.5						
Food cooked outdoors	1492	59.5						
								
*Number of rooms*			*NS*	*0.185*	*NS*	*0.595*	*NS*	*0.259*
1	855	34.1						
2	1168	46.6						
3	263	10.5						
*4*	128	5.1						
5	58	2.3						
>5	32	1.4						
								
*Number of occupants*				***0.001***	*NS*	*0.234*	*NS*	*0.381*
1	326	13.0	**1.08 [1.04–1.12]**	**<0.001**				
2	488	19.4						
3	529	21.1						
*4*	412	16.4						
5	281	11.2						
>5	473	18.9						
								
*Number of nets *^c^			*NS*	*0.088*	*NS*	*0.362*	*NS*	*0.192*
0	589	18.3						
1	1913	59.5						
2	548	17.1						
3	124	3.9						
4	26	0.8						
5	9	0.3						
>5	5	0.2						
								
*Type of net*			*NS*	*0.77*	*NS*	*0.693*	*NS*	*0.211*
None	590	18.4						
Zuia Mbu	1419	44.2						
Provided by the study for light trapping	221	6.9						
Other	984	30.6						
								
*Treatment status of net*^c^			*NS*	*0.486*	*NS*	*0.094*	*NS*	*0.362*
Treated in last six months	171	4.7						
Treated more than 6 months ago	239	6.6						
Never treated	3209	88.7						
								
*Holes >2 cm in net*			*NS*	*0.929*	*NS*	*0.296*	*NS*	*0.420*
0	1322	41.1						
1–5	838	26.1						
>5	3214	32.8						
								
*Net sides tucked in*			*NS*	*0.719*	*NS*	*0.541*	*NS*	*0.945*
0	774	24.1						
1–3	38	1.2						
4	2402	74.7						
								
*Occupants sleeping without net*^c^				***0.007***	*NS*	*0.083*	*NS*	*0.227*
0	1389	55.4	**0.93 [0.90–0.97]**	**<0.001**				
1	270	10.8						
2	257	10.2						
3	223	8.9						
4	139	5.5						
5	88	3.5						
>5	143	5.7						
								
*Additional people sleeping in trap room*			*NS*	*0.933*	*NS*	*0.281*	*NS*	*0.981*
0	1303	51.9						
1	173	6.9						
2	416	16.6						
3	381	15.2						
4	173	6.9						
5	44	1.8						
>5	19	0.7						
								
*Other protection against mosquitoes*			*NS*	*0.373*	*NS*	*0.354*	*NS*	*0.692*
None	2475	98.6						
Sprays, coils, herbal, physical	34	1.4						

^a ^Total number of times this value was recorded irrespective of whether repeated sampling the same houses was conducted.

^b ^As determined using stepwise selection of mixed linear models considering repeated sampling and cluster-round specific effects as random factors (See methods). The significance of each factor is presented as initially determined using the full model. The influence of factors confirmed to be significant by stepwise model selection are presented as the odds ratio for each level of that factor or, in the case of continuous variables, the odds ratio per unit increase and the significance as estimated using the final selected model. -2 Restricted Log Likelihood for the final fitted models are 1180, 868 and 1245 for *An. gambiae s.l*., *An funestus *and *Culex *species, respectively.

^c ^Under normal condition before any provision of a bednet to allow light trap sampling of mosquitoes.

NS Not significant.

NA Not applicable.

ND Not determined.

Other than bednets, closed eaves and window screening, almost no additional personal or household protection measures were used in the sampled houses and these measures had no detectable influence on indoor mosquito densities. The number of occupants and the number of occupants unprotected by bednets had modest but independently significant and opposite effects on the numbers of *An. gambiae *caught in light traps (Table 3). As previously described [97], increasing numbers of occupants result in slightly increased mosquito biting densities per person, probably due to the increased attractiveness and range of the odour plume associated with the house [98,99]. Although the availability of unprotected hosts nearby slightly suppressed the sensitivity of the trap, this effect was relatively modest, indicating that the attractiveness of unprotected individuals is not much greater than someone in a bednet with a light trap. This observation is consistent with the low levels of net treatment in the area [92] and the modest excito-repellent activity of many modern pyrethroid formulations [93,94]. Neither the number of nets in the house, type of net, treatment status of net, number of holes in net nor number of sides of the net that were tucked in had any significant effect on light trap catches of *An. gambiae*, *An. funestus *or *Culex *species. Indeed, apart from the effect of wall construction on *An. funestus*, catches of both *An. funestus *and *Culex *species appear to be uninfluenced by any of the recorded characteristics of the sampled houses. In agreement with another study in West Africa [73,100], CDC light traps are a relatively robust sampling tool for measuring mosquito densities in houses in the Kilombero Valley, even in the presence of bednets which may be treated.

### Bednet coverage, age distribution and personal protection

**Figure 5:**
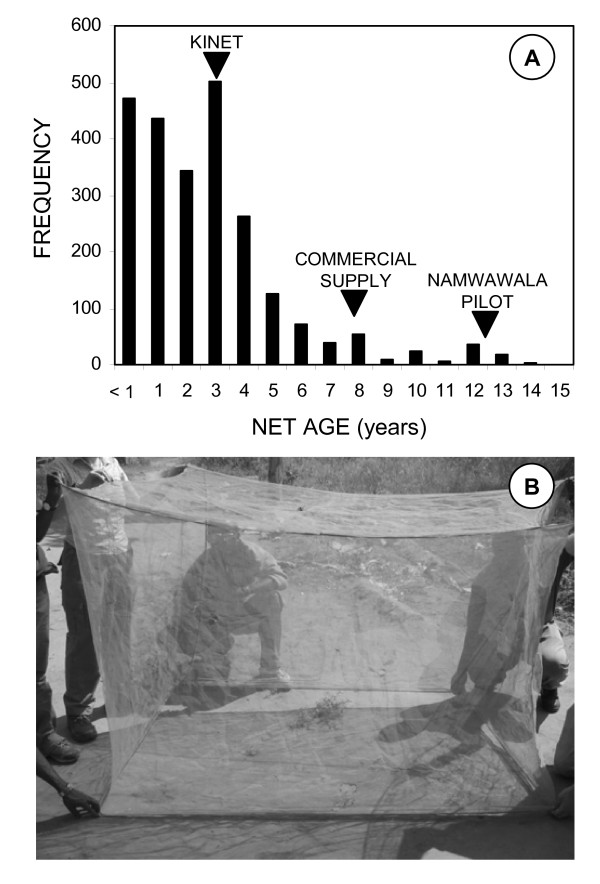
The frequency distribution of the age of nets in houses sampled for mosquitoes (**A**) and (**B**) a 15-year old polyethylene net still in use in Namwawala village, July 2004. This net was verified to still be in use at the time of publication, 18 years after initial distribution.

The use of bednets has been linked to the densities of non-vector *Culex *sp. mosquitoes but such nuisance mosquitoes appear to be, if anything, less abundant in Kilombero than in the urban Dar es Salaam where this relationship was described [101]. We therefore have no reason to assume net promotion was particularly easier in rural Kilombero because of intense nuisance biting and suggest such approaches may be broadly applicable in a variety of settings with appreciable mosquito densities. Interestingly, the frequency of bednet ages in surveyed houses revealed some important historical features of the quality and quantity of nets delivered in the Kilombero Valley (Figure 5). A large number of nets purchased at the start of the KINET social marketing programme [41], were still in use 3–4 years later (Figure 5a). Even a small number purchased when untreated nets first became commercially available remain in use. Most surprisingly, 63 nets that were 10 or more years old were found to be still in use. Two thirds of these (42) were found in Namwawala village where the first early pilot trials of nets were conducted *circa *1990 using polyethylene nets (SiamDutch Company). We traced a number of these nets in 2004 to verify their existence and examine their condition (Figure 5b). Generally, these nets were in remarkably good condition as they had no more holes >2 cm than polyester nets in use for 5 years or less and far less than those between 6 to 9 years old (Mean number of holes per net = 7.7 ± 0.22, 11.3 ± 1.09 and 8.8 ± 1.36 for nets ≤ 5, 6–9 and ≥ 10 years old, ANOVA d.f.= 2400, F = 7.33, P = 0.001; The only significant difference between the groups was between the first and second group, P < 0.001 by *post hoc *Least Significant Difference test). Our results confirm previous field studies [92] indicating that most nets in Kilombero are not treated (Table 3) and unlikely to possess satisfactory levels of insecticidal or excitorepellent activity. Nevertheless, we combine these entomological surveys with recent estimates of personal protection [69] to estimate the EIR experienced in each cluster by the minority of residents who used a well-maintained and treated ITNs (Table 2), indicating these individuals experience far lower mean exposure (Table 2).

### Effects of net coverage on malaria transmission intensity

**Figure 6:**
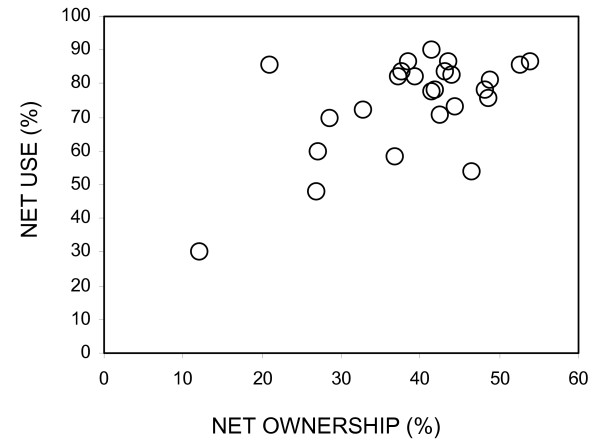
Relationship between reported net usage (proportion of people sleeping under nets) and net ownership (number of nets per person).

**Figure 7:**
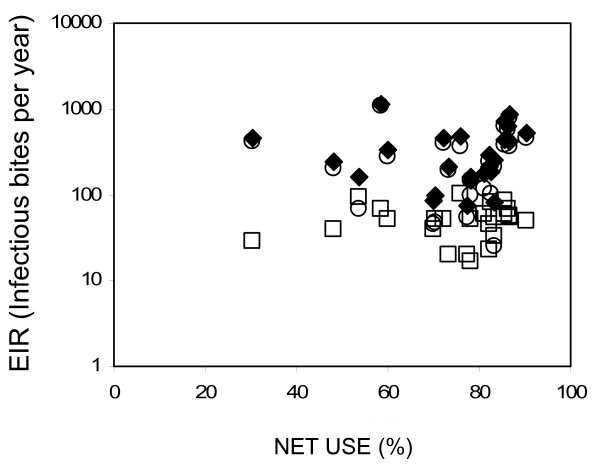
Relationship between reported net usage (proportion of people sleeping under nets) and Entomological Inoculation Rate (EIR) experienced by non users and net usage (open circles: *An. gambiae*; open squares: *An. funestus*; solid diamonds: Total).

Although reflecting quite different measures, the two indicators of net coverage based on ownership (number of nets owned divided by the total number of members in surveyed households) and usage (number of people reporting net use the previous night divided by total number of people surveyed) were closely related (Pearson's correlation r = 0.586, P = 0.002; See Figure 6). Net ownership per person in the surveyed subvillages ranged from 12 to 53 % and net usage ranged from 30 to 90%. Overall, the mean net occupancy was 1.9 persons per net with coverage in terms of usage exceeding 50% in all but two clusters. Net occupancy declined with net ownership (r = -0.695, P = 0.748), reflecting reduced necessity for individuals to share the protection of available nets and saturation at high coverage levels (Figure 6). Overall, these represent quite high levels of coverage and a substantial continuing improvement upon previous reports [41], indicating that net acquisition and use was sustained beyond the end of the KINET project. However, EIR mediated by neither vector species nor by their combined total was correlated to either indicator of net coverage, whether aggregated by cluster or village (Figure 7, Table 4).

**Table 4:** Correlations between bednet coverage and entomological inoculation rate.^a^

Outcome parameter^b^	Net Coverage^c^	
	
	Correlation Coefficient	P
Entomologic inoculation rate (EIR)		
Non users		
*Anopheles gambiae s.l*.	-0.068	0.747
*Anopheles funestus*	0.035	0.867
Total	-0.063	0.763

^a ^Estimated by Pearson's correlation. Results of Spearman's rank correlation were essentially identical and not shown.

^b ^See table 2.

^c ^Based on net usage as an indicator of coverage and aggregating by cluster. Analyses based on net ownership estimates and aggregated at village level yielded essentially identical results.

**Figure 8:**
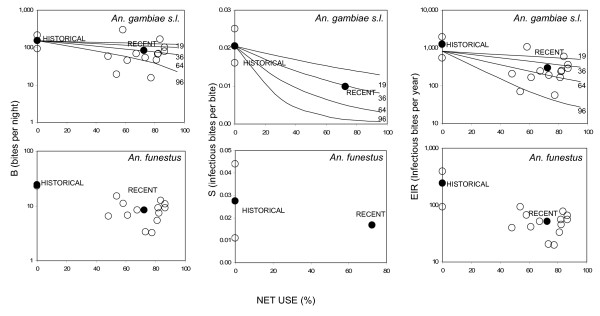
Relationship between bednet coverage and malaria transmission. Field observations of village-level human biting rates (B), sporozoite prevalence (S) and entomological inoculation rate (EIR) *An. gambiae s.l*. and *An. funestus *are plotted as a function of recent (October 2001–September 2003) and historical (early to mid 1990's with reported bednet use rates approximating zero). Note that B and EIR reflect community-level means values for non-users of bednets and recent values of S can only be reported as an overall mean for the entire study area. Open circles: Individual villages included in recent surveys, filled circles: mean of all villages historically or recently. For *An. gambiae s.l*., expected trends based on simulation modelling is presented as continuous lines for bednets which confer 19, 36, 64 and 96 % protection against indoor exposure (See methods).

This surprising lack of an apparent relationship between bednet coverage and community-level malaria transmission intensity is, however, readily explained when taken in the context of historical trends (Figure 8, Table 5), the extreme spatial heterogeneity of malaria transmission intensity [55,102-106], and the smoothing effect that mosquito dispersal has upon locally variable impacts of interventions coverage [18,31,107,108]. First of all, when considered at village scale to minimize the spatial smoothing effects of mosquito dispersal upon impact, we see that reasonably high coverage is achieved in all villages and that between-village variation in EIR is at least equivalent to that which might be expected from such modest variations in coverage with nets of generally poor quality (Figure 8). This is consistent with observations in previous bednet trials showing enormous variations between villages in the same study area, requiring large sample sizes to demonstrate impact even where coverage is deliberately manipulated to be systematically heterogeneous [18,19,24-26,108,109]. Fortunately, some previous reports from the study area allow approximate comparison with historical norms, before the introduction of bednets (Table 5, figure 8). In both villages for which historical data exists, biting rates are substantially lower than previously for both vector species. Interestingly, recalculation of historical EIR values so that they are comparable with these more recent estimates, resulted in exceptionally high values (Table 5, figure 8) which are more than double those originally published [54,62,65]. While this dramatic change is to some degree the result of using absolute, rather than geometric means, this is primarily caused by using a site-specific estimate of light trap efficiency rather than an external estimate from northern Tanzania. While we were unable to make village-specific estimates of sporozoite prevalence, the overall mean reported here at high bednet coverage is substantially lower than previous reports for both vector species in both villages previously surveyed. In both villages for which estimates are historically available, EIR appears to have been substantially reduced. Overall, this crude estimate of impact on community-level EIR, which is enjoyed by user and non-users alike, is approximately equivalent to the personal protection provided by actually using a reasonably maintained ITN [69]. Furthermore, these crude comparisons are approximately consistent with previous observations that even untreated nets can not only provide personal protection [110] but also suppress communal malaria transmission [20]. In fact both recent epidemiological evidence from this study site [23] and simulations of such coverage levels with bednets conferring modest personal protection levels against 30–60% of exposure (Figure 8) indicate that in this case, where insecticide treatment levels are low (Table 3 and [92]), communal protection exceeded personal protection.

**Table 5:** Village-level estimates of recent^a ^and historical^b ^malaria transmission intensity.

Village	Recent bednet use (%)	Species	Biting Rate (bites per person per night)			Sporozoite Prevalence (infectious bites per bite)			Entomologic Inoculation Rate (infectious bites per person per year)			References
						
			Historical	Recent	Difference	Historical	Recent	Difference	Historical	Recent	Difference	
												
Idete	86.5	*An. gambiae*	211	80	-62	0.0251	0.0098^c^	-61	1931	285	-85	[65]
		*An. funestus*	25	11	-56	0.0440	0.0167^c^	-62	394	66	-83	
		Total							**2326**	**350**	**-85**	
												
Iragua	58.3	*An. gambiae*		296			0.0098^c^			1059		
		*An. funestus*		11			0.0167^c^			67		
		Total								1126		
												
Kichangani	48.2	*An. gambiae*		57			0.0098^c^			206		
		*An. funestus*		6			0.0167^c^			39		
		*Total*								**245**		
												
Kidugalo	53.9	*An. gambiae*		19			0.0098^c^			69		
		*An. funestus*		15			0.0167^c^			93		
		Total								**162**		
												
Kivukoni	81.2	*An. gambiae*		47			0.0098^c^			167		
		*An. funestus*		5			0.0167^c^			33		
		Total								**200**		
												
Lukolongo	86.5	*An. gambiae*		100			0.0098^c^			358		
		*An. funestus*		9			0.0167^c^			56		
		Total								**414**		
												
Lupiro	83.1	*An. gambiae*		165			0.0098^c^			590		
		*An. funestus*		13			0.0167^c^			77		
		Total								**667**		
												
Mavimba	81.9	*An. gambiae*		67			0.0098^c^			236		
		*An. funestus*		9			0.0167^c^			56		
		Total								**295**		
												
Mbingu	73.3	*An. gambiae*		54						192		
		*An. funestus*		3						20		
		Total								**212**		
												
Mchombe	82.2	*An. gambiae*		69						247		
		*An. funestus*		7						45		
		Total								**292**		
												
Minepa	77.5	*An. gambiae*		15			0.0098^c^			55		
		*An. funestus*		3			0.0167^c^			20		
		Total								**75**		
												
Mkangawalo	61.2	*An. gambiae*		46			0.0098^c^			163		
		*An. funestus*		7			0.0167^c^			41		
		Total								**204**		
												
Namwawala	67.5	*An. gambiae*	93	68	-26	0.0160	0.0098^c^	-39	545	245	-55	[54, 62]
		*An. funestus*	23	8	-63	0.0110	0.0167^c^	-52	92	51	-44	
		Total							**637**	**296**	**-53**	
												
Overall	72.4	*An. gambiae*	152	83	-44	0.0245	0.0098^c^	-60	1238	298	-70	
		*An. funestus*	24	8	-59	0.0196	0.0167^c^	-15	243	51	-63	
		Total							**1481**	**349**	**-69**	

^a ^As estimated from the October 2001–September 2003 surveys described here.

^b ^All previous reports from Kilombero and Ulanga districts except for the two villages of Michenga [86] and Kibaoni [53] which were not included in this study, as well as two reports from Ifakara town [52,87] which are considered urban or per-urban and therefore cannot be rationally compared with any of the other sites surveyed [88-90].

^c ^Separate village-specific sporozoite infection prevalence estimates could not be obtained from the recent surveys because of lack of sufficient samples from each village. Recent sproozoite prevance is therefore presented as a common overall estimate for the entire study area, including all villages.

An important point to bear in mind is that those actually using an effective ITN receive both personal and communal protection. The mean EIR experienced by an ITN user in Kilombero during the study period is estimated to be 105 infectious bites per person per year (Table 2), over an order of magnitude lower than historical norms before ITNs became available and popular (Table 5). At the time these surveys were conducted, few residents enjoyed the benefits of well-treated and maintained nets [92] so, consistent with epidemiological reports [23,41], personal protection probably contributed less protection to the average user than the communal protection reported here or that expected from a truly insecticidal net. Nevertheless, even if we consider a conservative estimate of protection against 40% of bites, consistent with Figure 8 and previous entomological evaluations of untreated nets [111-114], we estimate that a typical net user experienced an EIR of 210 infectious bites per year. Weighting estimated exposure for users and non-users by the proportion of the population they comprise, we estimate that the overall mean EIR for all net users and non-users was 244 infectious bites per person per year. While these are high transmission intensities by any standards, these nevertheless represent 93, 86 and 83% reductions relative to historical norms for an ITN user, an average net user and an average resident of the valley respectively. Given that the impacts of transmission-reducing interventions should be considered multiplicatively along a linear scale [115,116] or additively along a logarithmic scale [83,84], we present these impacts in terms of fold reduction relative to historical norms: Although malaria transmission remains intense in Kilombero, exposure has been reduced by approximately 4-fold for non-users of nets, 6-fold for the average resident, 7-fold for users of typical nets and 14-fold for users of truly insecticidal nets, when compared with an exceptionally high historical mean of 1481 infectious bites per person per year.

## Discussion

This study represents the first area-wide evaluation of malaria transmission and the impacts of high coverage with nets upon it in the Kilombero Valley. Bednets are now commonplace in this area and coverage levels in the whole population, rather than just target groups, exceeded the thresholds required to achieve community-level suppression of transmission with insecticidal nets [14,16]. Overall, the valley remained an area of intense malaria transmission because of extremely high seasonal abundance of both *An. gambiae *and *An. funestus*. Nevertheless, comparison with historical data indicates that transmission intensity was approximately four fold higher a decade previously and that substantial reductions of community-level transmission were attained even though the bednet technologies available at the time were very poor and are now considered obsolete [5,15,117]. Notably, the 75% net usage attained across all age groups in Kilombero Valley by 2004 compares very well with that recently attained amongst young children through targeted mass distributions to "catch up" and subsidized sales to "keep up" in Kenya (81% [34]) and Ghana (73% [12]). It is particularly remarkable that the public-private hybrid delivery system described here was supported with quite modest subsidies and correspondingly recovered most of the costs of provision to the population as a whole and even the vulnerable groups to whom subsidy was particularly targeted. For example, the Tsh 500 (approximately US$0.84 at the time) voucher subsidy provided by the KINET programme comprised only 15% of the full delivery cost. This level of subsidy was substantially less than the Tsh 2750 (approximately US$2.15 at the time of submission) voucher subsidy currently provided by the Tanzanian National Voucher Scheme [51] or the ≥$2.00 subsidies of nets sold to sustain coverage in Kenya [34] and Ghana [12].

Our crude estimate of 69% reduction of EIR for non-users of ITNs (Table 5, Figure 8) is very similar to previous estimates of 70–73% for ITNs in the same area [69] which presumably explain the reduced morbidity and mortality of ITN users[41,42]. Although the results presented does not constitute probable evidence for community-level suppression of transmission, often referred to as the "mass effect", it does present a very plausible case [32] that such equitable alleviation of malaria burden [14,16] is both important and achievable with subsidized ITN promotion approaches such as the Tanzanian National Voucher Scheme [51]. These observations are consistent with epidemiological evaluations at finer scales in the village of Idete, demonstrating protection of both users and non-users against anaemia and splenomegaly by high net coverage in their immediate surroundings [23]. It is also noteworthy that the mean level of personal protection afforded by the typical standard of bednet used in Kilombero is approximately matched by that observed for closed eaves (Table 3). It may therefore be possible to achieve similar levels of communal protection with this much neglected intervention option [96,118] if readily achievable changes in housing structure could be promoted in the area.

The simulations presented in Figure 8 and similar analyses published elsewhere [14,91,115] also suggest that ITN promotion strategies could achieve massive reductions of malaria exposure if the same coverage levels were attained with new longer-lasting ITN technologies [117]. In Tanzania, as in other settings, home-based re-treatment of nets with insecticide is difficult to achieve [45], resulting in very low levels of coverage (Table 3 and [92]) unless provided during free re-treatment campaigns [119,120]. Consistent with recent epidemiological reports [23], here we report communal protection as a result of high coverage with poor quality nets which appears greater than the personal protection afforded to individual users. Importantly, community-level protection is directly related to the coverage and level of personal protection [14,91,115]. Thus if rapidly improving levels of personal protection with ITNs [93-95] could be achieved at the coverage levels demonstrated here, we expect that the exposure of non-users and users would be reduced by at least one and two orders of magnitude, respectively (Figure 8). The observation of intact polyethylene nets still in use after up to 18 years further supports the case for prioritizing improvements in the quality as well as quantity of nets in use. Indeed some long-lasting ITN products can continue killing mosquitoes after up to 8 years of use [119] and are indistinguishable from factory-fresh samples in experimental hut trials after 4 years of typical village use [94]. New long-lasting treatment kits [121] may allow rapid and permanent impregnation of nets already in use, including the cheaper polyester nets which are commonly available across much of Africa today. Long-lasting insecticide-treated nets represent a promising means to achieve high levels of coverage with insecticide treatment [117] and realize the full communal and personal protection of ITNs in communities across Africa [5,14,16,34,91,115].

We nevertheless caution that theoretical projections should be interpreted cautiously if historical mistakes [122] are not to be repeated. While encouraging, the projected impacts of combining this particular promotion strategy with improved ITN technology (Figure 8) should be interpreted critically, considering three essential caveats: 1) Substantially greater subsidies will be required to make excellent but more expensive long-lasting polyethylene nets affordable through cost-sharing systems such as the one described here. 2) Despite the challenges of doing so [31], the complex personal and communal benefits of increasing coverage with ITNs with varying quality should be continually evaluated through rigorous field studies. 3) While huge reductions of human exposure to malaria are possible with increasing coverage of ITNs, the intense transmission levels which commonly occur in Africa are unlikely to be completely addressed with any single intervention. We therefore suggest that as national programmes strive to alleviate malaria burden in resource-poor countries across Africa [3,4], the quantity, quality and benefits of ITNs are continually evaluated and augmented with complementary interventions which target all stages of the vector and parasite life cycles.

## Conclusion

A cost-sharing scheme which combines largely private sector distribution with limited but targeted public subsidies has achieved sustained coverage of 75% bednet use across all age-groups in a large rural population in southern Tanzania. Despite the generally poor quality and treatment standards of these nets, community-level protection was achieved that is approximately equivalent to the personal protection of a typical ITN. Furthermore, even greater and more equitable gains for net users and non-users are anticipated if long-lasting ITNs can be similarly promoted with augmented subsidies to cover the extra cost of these more expensive technologies. The World Health Organization's latest position statement [5] emphasizes that free or highly subsidized mass distribution of ITNs is now considered a proven strategy [8,12,34]. However, in contrast to some recent suggestions [15], this recommendation does not exclude alternative approaches which may be equally successful in specific contexts [5]. Furthermore, we caution that the evidence base supporting the clear success of highly subsidized mass distribution relies exclusively on coverage of vulnerable population groups only [8,12,34] and therefore falls short of demonstrating potential to achieve communal protection [14]. Here we show for the first time that "keep up" programmes relying exclusively on sales of modestly subsidized nets can achieve and sustain high coverage of entire populations with bednets, even without any complementary "catch up" mass distribution component. As the world considers the true scale of financial commitment required to effectively tackle malaria [33], such cost-sharing schemes for ITN delivery represent an important option for governments, NMCPs and donor partners in Africa. For now, there simply isn't enough money available to NMCPs to address all their needs and current international commitments total only 20% of what is actually required [33]. In Africa alone, a minimum of US$1.7 billion will be required annually to support all essential malaria control activities in the coming years. Approximately US$680 million per annum, or 40% of this grossly underfinanced need, will be required for fully subsidized vector control, primarily ITNs and indoor residual spraying [33]. While cost sharing certainly can limit coverage of the poorest with personal protection [34,48], the more important communal protection delivered by high net coverage is, by definition, completely equitable and comprehensive[14]. Any delivery strategy which enables consensus coverage targets for ITNs across all age groups [5,14] to be achieved with limited public subsidies therefore merits careful consideration. We conclude that the cost sharing approach described here represents a valid, effective and important option for NMCPs currently faced with huge gaps between their operational ambitions and the financial resources at their disposal.

## Competing interests

The author(s) declare that they have no competing interests.

## Authors' contributions

GFK provided partial supervision towards the end of the field and laboratory data collection, assembled, cleaned and analyzed the data and wrote the manuscript in consultation with all the other authors. AT supervised most of the field work, initiated laboratory analysis and participated in interpretation of the results and writing of the manuscript. JK supervised all aspects of data collection in the field and assisted with interpretation of the results. FRO-O and MEK co-supervised the calibration of the sampling methods and contributed to the drafting of the manuscript. HG contributed to collection, assembly and interpretation of all geographic data. NK and HN collected the bulk of the entomological and interview data and assisted in data collation, cleaning and interpretation. VM coordinated and implemented all aspects of the laboratory analysis and contributed to the drafting of the manuscript. RN and SA helped design the study, coordinated the collection of demographic and bednet coverage data, interpretation of the results and drafting of the manuscript. JDC contributed to the design of the study, the interpretation of the data and the drafting of the manuscript. TAS and CL designed the study and contributed substantially to the analysis, interpretation and drafting of the manuscript. All authors read and approved the final manuscript

## Pre-publication history

The pre-publication history for this paper can be accessed here:


